# Transcriptome Analysis Reveals Key Flavonoid 3′-Hydroxylase and Flavonoid 3′,5′-Hydroxylase Genes in Affecting the Ratio of Dihydroxylated to Trihydroxylated Catechins in *Camellia sinensis*

**DOI:** 10.1371/journal.pone.0137925

**Published:** 2015-09-14

**Authors:** Kang Wei, Liyuan Wang, Chengcai Zhang, Liyun Wu, Hailin Li, Fen Zhang, Hao Cheng

**Affiliations:** 1 National Center for Tea Improvement, Tea Research Institute Chinese Academy of Agricultural Sciences (TRICAAS), 9 Meiling South Road, Hangzhou, Zhejiang 310008, China; 2 Key Laboratory of Tea Plant Biology and Resources Utilization, Ministry of Agriculture, Hangzhou 310008, China; NBPGR, INDIA

## Abstract

The ratio of dihydroxylated to trihydroxylated catechins (RDTC) is an important indicator of tea quality and biochemical marker for the study of genetic diversity. It is reported to be under genetic control but the underlying mechanism is not well understood. Flavonoid 3′-hydroxylase (F3′H) and flavonoid 3′,5′-hydroxylase (F3′5′H) are key enzymes involved in the formation of dihydroxylated and trihydroxylated catechins. The transcriptome and HPLC analysis of tea samples from Longjing43 and Zhonghuang2 under control and shading treatment were performed to assess the F3′H and F3′5′H genes that might affect RDTC. A total of 74.7 million reads of mRNA seq (2×101bp) data were generated. After *de novo* assembly, 109,909 unigenes were obtained, and 39,982 of them were annotated using 7 public databases. Four key F3′H and F3′5′H genes (including *CsF3′5′H1*, *CsF3′H1*, *CsF3′H2* and *CsF3′H3*) were identified to be closely correlated with RDTC. Shading treatment had little effect on RDTC, which was attributed to the stable expression of these key F3′H and F3′5′H genes. The correlation of the coexpression of four key genes and RDTC was further confirmed among 13 tea varieties by real time PCR and HPLC analysis. The coexpression of three F3′H genes and a F3′5′H gene may play a key role in affecting RDTC in *Camellia sinensis*. The current results may establish valuable foundation for further research about the mechanism controlling catechin composition in tea.

## Introduction

**Fig 1:**
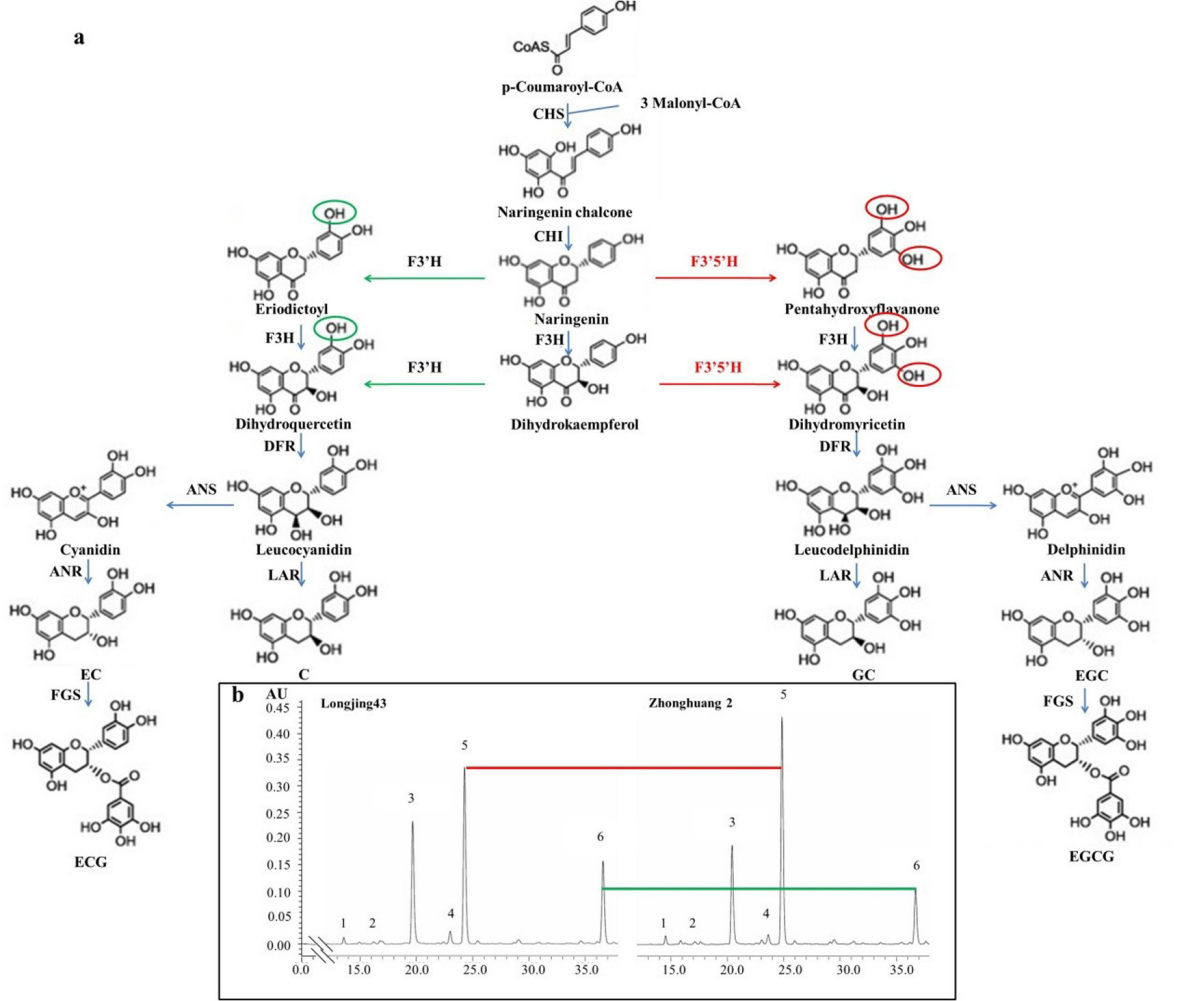
Catechin biosynthetic pathway and chromatogram of typical tea samples. a.Catechin biosynthetic pathways in *Camellia sinensis*.F3′H and F3′5′H are two key enzymes controlling the hydroxylation of naringenin and dihydrokaempferol at either the 3′ position or at both the 3′ and 5′ positions in the B ring. Abbreviations of enzymes are as follows: CHS, chalcone synthase (EC 2.3.1.74); CHI, chalcone isomerase (EC 5.5.1.6); F3H, flavanone 3-hydroxylase (EC 1.14.11.9); F3′,5′H, flavonoid 3′,5′-hydroxylase (EC 1.14.13.88); F3′H, flavonoid 3′-hydroxylase (EC 1.14.13.21); FLS, flavonol synthase (EC 1.14.11.23); DFR, dihydroflavanol 4-reductase (EC 1.1.1.219); ANS, anthocyanidin synthase (EC 1.14.11.19); ANR, anthocyanidin reductase (EC 1.3.1.77); LAR, leucocyanidin reductase (EC 1.17.1.3); FGS, flavan-3-ol gallate synthase (EC number not assigned). b. Chromatogram of tea samples from Longjing43 and Zhonghuang2 under control. Peak 1, EGC; 2, C; 3, Caffeine; 4, EC; 5,EGCG; 6,ECG. Zhonghuang2 had higher EGCG content and Longjing43 had higher ECG content.

Tea (*Camellia sinensis* L.) is a popular drink worldwide, and it is purported to offer protection against cancer [1], cardiovascular diseases [2] and obesity-related disorders [3]. These benefit effects are mainly associated with the high accumulation of catechins, which possess strong radical scavenging and antioxidant effects [4]. In fact, tea catechins have ideal structural chemistry for free radical scavenging activities. They appear to be more effective antioxidants *in vivo* than vitamins E or C [5]. Major catechins in tea include (-)-epigallocatechin gallate (EGCG), (-)-epigallocatechin (EGC), (-)-epicatechin gallate (ECG), (-)-epicatechin (EC), (-)-gallocatechin (GC) and (+)-catechin (C). Among them, EGCG was found to be the most abundant and effective scavenger of free radicals [6]. According to their structural differences at the 5′ position in the B ring, tea catechins can be divided into dihydroxylated (ECG, EC and C) and trihydroxylated catechins (EGCG, EGC and GC) (Fig 1A). Structure-activity relationship studies have shown that the presence of the hydroxyl group at the 5′ position in the B ring is crucial for scavenging activity. For example, the free radical scavenging effects of EGC and GC are stronger than those of EC and C [7,8]. Furthermore, the ratio of dihydroxylated to trihydroxylated catechins (RDTC) in *C*. *sinensis* is reported to be under genetic control and widely used as an indicator of tea quality and as a biochemical marker for studying genetic diversity [9–13]. It is therefore highly essential to understand the underlying mechanism controlling the RDTC in *C*. *sinensis*, which still remains unclear.

Tea catechins are biosynthesized by the phenylpropanoid and flavonoid pathways, in which dozens of enzymes are involved [14]. Flavonoid 3′-hydroxylase (F3′H) and flavonoid 3′,5′-hydroxylase (F3′5′H) are two important enzymes controlling the hydroxylation of naringenin and dihydrokaempferol at either the 3′ position or at both the 3′ and 5′ positions in the B ring (Fig 1A) [15]. The resultant intermediates then flow through downstream enzymes to form dihydroxylated and trihydroxylated flavonoids. In this way, F3′Hs and F3′5′Hs play a key role in affecting the composition of dihydroxylated and trihydroxylated flavonoids. For example, Castellarin et al. (2006) reported that the expression of F3′H and F3′5′H genes directly affected the accumulation of cyanidin-(dihydroxylated)/delphinidin-(trihydroxylated) based anthocyanins in berry skin of grapevines, which determines the color variation among grape varieties [16]. However, to date, no correlation between the expression of F3′H and F3′5′H genes and RDTC has been established in *C*. *sinensis*. One of the reasons for this is that there may be many F3′H and F3′5′H genes in *C*. *sinensis* and the key genes have not yet been characterized. Another reason is that RDTC might be dependent on the coexpression of both F3′H and F3′5′H genes rather than either type alone, which renders previous correlation analyses of candidate gene expressions and catechin contents difficult to unravel [17,18].

The RNA-Seq method, which was recently developed, largely overcomes these limitations [19–21]. RNA-Seq method can be used to identify genes that may encode F3′H and F3′5′H enzymes and so identify the key genes according to their expressions. Moreover, the combination of metabolome and RNA-Seq analyses can provide precise information regarding gene-to-metabolite networks for identifying key genes. For example, Wu et al. (2014) reported six catechin biosynthetic genes closely associated with their corresponding catechins in *C*. *sinensis* mainly by the combination of HPLC and RNA-Seq analyses [22]. Therefore, identification of the association between RDTC and RNA-Seq results can largely facilitate relative studies. On the other hand, a novel chlorophyll-deficient chlorina tea cultivar, Zhonghuang2 was recently bred by Tea Research Institute Chinese Academy of Agricultural Sciences (TRICAAS), which showed lower total catechin contents than the normal green tea cultivar Longjing43 [23]. Further characterization of the individual catechins showed the RDTC in Zhonghuang2 to be much lower than in Longjing43 (Fig 1B). Because shading treatment facilitates chlorophyll formation and largely affects catechin biosynthesis, it was also used to determine its effect on RDTC. Shading treatment largely decreased all catechin contents, but it had little effect on RDTC in either cultivar, which raises questions about how the RDTC was regulated. In this study, tea samples from both cultivars under control and shading treatment were used for RNA-seq analysis (NCBI BioProject Accession: PRJNA261659, http://www.ncbi.nlm.nih.gov/bioproject/261659) and HPLC analysis of catechins. The purposes of this work were (1) to identify key F3′H and F3′5′H genes affecting RDTC in *C*. *sinensis*; (2) to investigate variety and shading effects on RDTC in *C*. *sinensis*.

## Materials and Methods

### Plant Material

Tea cultivars ‘‘Longjing 43″ and “Zhonghuang 2”were grown in the experimental tea garden of TRICAAS in Hangzhou, China (120°12′E, 30°16′N). Each tea field was separated into two parts, one subjected to the shading treatment with sun shade net (2 m × 20 m, shading rate: 80 ± 5%) and the other one without shading (control). The shading experiment was conducted from July 1st to July 7th 2013. After that, fresh materials (one leaf and a bud) were collected from each treatment and stored at -70°C. Parts of tea samples were used for RNA-Seq experiment. The rest tea samples were dried by a freeze dryer (Labconco Stoppering Tray Dryer, Labconco Inc., Kansas City, MO) at -5°C for 72 hours. The freeze-dried samples were then subject to catechin analysis.

Furthermore, thirteen tea varieties, namely Longjing43, Zhonghuang2, Fudingdabaicha, Anjibaicha, Wuniuzao, Zhongcha108, XKW15, Qiqu3, Lj001, XKW8, Xinxuan7, XKW10, XKW5 grown in the experimental tea garden of TRICAAS were utilized for verification experiment. Fresh materials (one leaf and a bud) were collected on Sep 2, 2014 from each variety and stored at -70°C. Parts of tea samples were used for real time PCR analysis. The rest tea samples were dried by the freeze dryer and subject to catechin analysis.

### RNA Sequencing Analysis

RNA sequencing of four tea samples (control of Longjing 43, shading treatment of Longjing 43, control of Zhonghuang2, shading treatment of Zhonghuang2), *de novo* assembly and functional annotation were performed exactly according to the method described by Wei et al. (2014) [20]. All genes annotated to F3′H and F3′5′H were identified for further analysis. Furthermore, amino acid sequences of key F3′Hs (*CsF3′H1*, *CsF3′H2*, *CsF3′H3*) and *CsF3′5′H1* identified in this study as well as other F3′H, F3′5′H genes obtained from NCBI website were aligned using ClustalX. A Phylogenetic tree was constructed from the clustal alignment using the maximum-likelihood method in the MEGA5.05 package.

### Expression Analysis of Candidate F3′H and F3′5′H Genes

For expression analysis of candidate F3′H and F3′5′H genes, reads per kilobase per million reads (RPKM) was used as a value of normalized gene expression [24]. Statistical comparison of RPKM values among four tea samples was conducted using a web tool IDEG6 (http://telethon.bio.unipd.it/bioinfo/IDEG6_form/) [25].

### Catechin Analysis

#### Standard Chemicals

The standard chemicals, including caffeine, C, EC, EGC, EGCG, GC and ECG were purchased from Sigma Chemical Company (St. Louis, MO, USA).

#### Extraction Method

Tea sample (0.2 g, dry weight) was extracted with 5 ml 70% methanol in a water bath at 70°C for 10 min with intermittent shaking. The supernatant was then centrifuged at 3500 × g for 10 min at 4°C and transferred to a 10 ml volumetric flask. The extraction steps were repeated again to reach a final volume of 10 ml. The extracts were filtered through a 0.45-μm Millipore filter before the injection was made.

#### Chromatographic Analysis

The catechins contents of tea samples were analyzed through RP-HPLC according to the method described by Rio et al. (2004) [26]. The extracts were analyzed using an Agilent 1100 HPLC system. A Phenomenex RP-MAX C12 reverse phase column (4 μm 250 mm×4.6 mm i.d.) was used for chromatographic separation, which was maintained at 40°C, eluted at 1 ml min^-1^ with a 60 min gradient of 4–25% gradient of acetonitrile in water containing 1% formic acid and monitored at 280 nm [26]. RDTC was calculated by the formula as follows: RDTC = (ECG+EC+C)/(EGCG+EGC+GC).

### Real Time RT-PCR Analysis

To validate the correlation between the expressions of key F3′H and F3′5′H genes and RDTC in *C*. *sinensis*, *CsF3′5′H1* (comp149726_c0) and three key F3′H genes (*CsF3′H1*, comp143838_c0; *CsF3′H2*, comp147206_c0; *CsF3′H3*, comp141172_c0) were selected for real time RT-PCR analysis, using RNA isolated from tea samples from thirteen varieties. RNA isolation was done according to a modified CTAB method described by Chen et al. (2012) [27]. Primers were designed using Primer 3 software to amplify 200-241bp fragments for F3′Hs and 169 bp for *F3′5′H1* selected from the RNA-Seq libraries (Table 1). Real time expression assays were performed as described by Wei et al. (2014) [20]. The amplification efficiency (AE) was calculated as described by Hellemans et al. (2007) [28]. Relative amounts were calculated and normalized with respect to the internal control *GAPDH* transcript levels. The transcript levels for *CsF3′H1* and *CsF3′5′H1* in Longjing43 were taken as 1.0. Relative amounts of *CsF3′H2* and *CsF3′H3* were calculated according to the transcript level of *CsF3′H1* in Longjing43 as the method described by Tuteja et al. (2004) [29]. Expression ratios of F3′Hs to F3′5′H were calculated by the formula as (*CsF3′H1*+*CsF3′H2*+*CsF3′H3*)/ *CsF3′5′H1*.

**Table 1:** Primers used for quantitative real time RT-PCR.

Gene no.	Primer sequence(5′-3′)
*GAPDH(GE651107)*	F:TTGGCATCGTTGAGGGTCT
	R: CAGTGGGAACACGGAAAGC
*CsF3′H1(KP335091)*	F:GTACATTAGTGAATCCTTGCGTGAG
	R: AAAATCCTTCTCTTCCTCCTTCCTC
*CsF3′H2(KP347675)*	F:AAGCTCACTGACATCGAAATCAAAG
	R:AAGGTTAATTGGGCTAGGTCTGATT
*CsF3′H3(KP347676)*	F: TTAGAGCTTGCTGATGATCCTACTC
	R:CTTTTCTTCAACCCATCTATCTCGC
*CsF3′5′H1(KP347677)*	F: CCTACCCAAACTCCCATACTTACAA
	R: CAGGAATGAACTCTAATGGCCTTTC

### Data Analysis

Analysis of variance was performed with SPSS 16.0 statistical software. Differences among means were evaluated using the Duncan′s multiple range test [30]. Correlation coefficients were calculated for RDTC and expression ratios of F3′H genes to *F3′5′H1* gene in different varieties.

## Results

### Comparison of Catechin Contents in cv. Zhonghuang2 and Longjing43 Under Different Treatments

**Fig 2:**
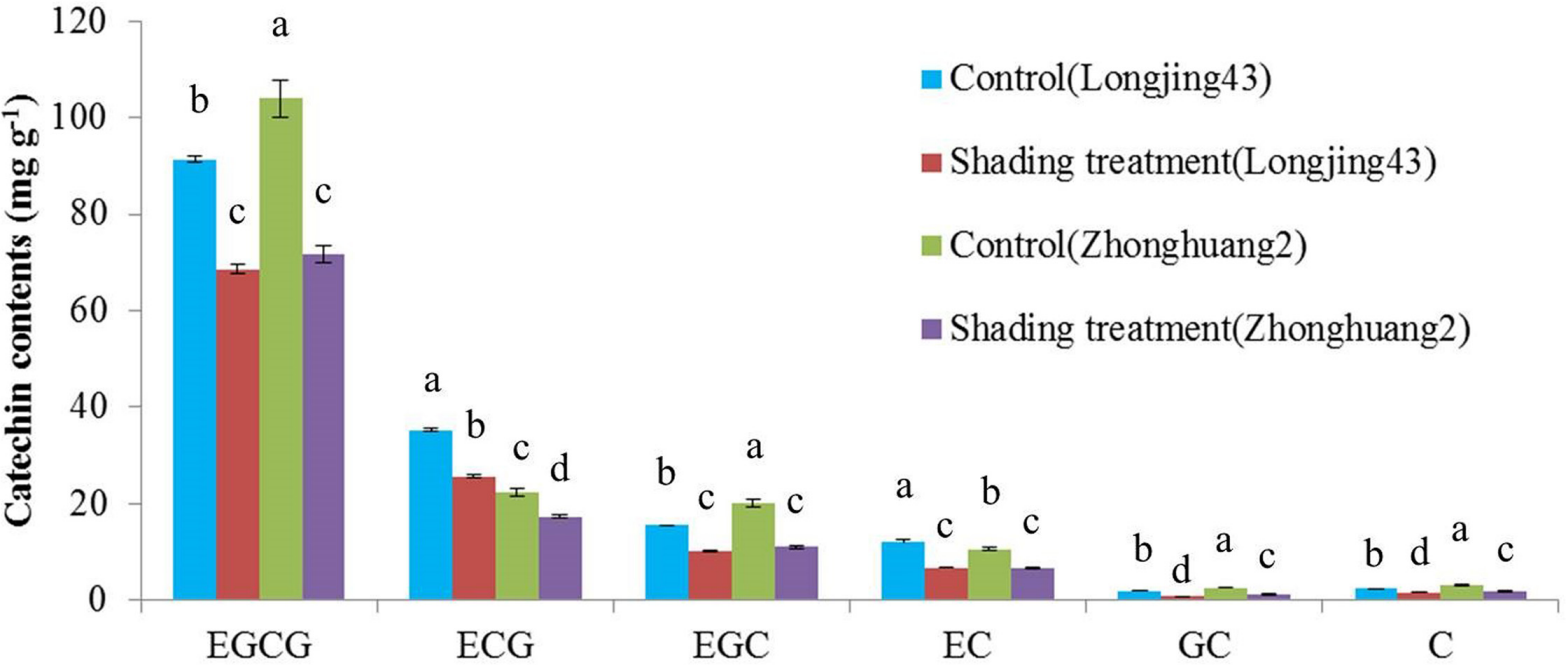
Catechin composition obtained by HPLC from Longjing43 and Zhonghuang2 under control and shading treatment (Mean±standard deviation, n = 3). Means in each column for each catechins labeled with the same letter are not significantly different (P>0.05) based on one-way ANOVA with Duncan′s multiple range test.

Typical chromatograms of catechins separated from controls of *cv*. Longjing43 and Zhonghuang2 are shown in Fig 1B, and means and standard deviations (SD) of six catechin contents in both cultivars under different treatments are shown in Fig 2. The HPLC results showed that EGCG, which ranged from 68.6 to 104.0 mg/g, was the most abundant catechin fraction, while GC was the least abundant. Shading treatment significantly decreased all catechin fractions, irrespective of cultivars. This is in agreement with previous reports [31, 32]. An interesting observation from this study is that the trihydroxylated catechin contents (EGCG, EGC and GC) of *cv*. Zhonghuang2 were significantly higher but the dihydroxylated catechin contents (ECG, EC and C) were significantly lower than those of Longjing43, irrespective of treatments, indicating the RDTC of Zhonghuang2 is much lower than that of Longjing43.

**Fig 3:**
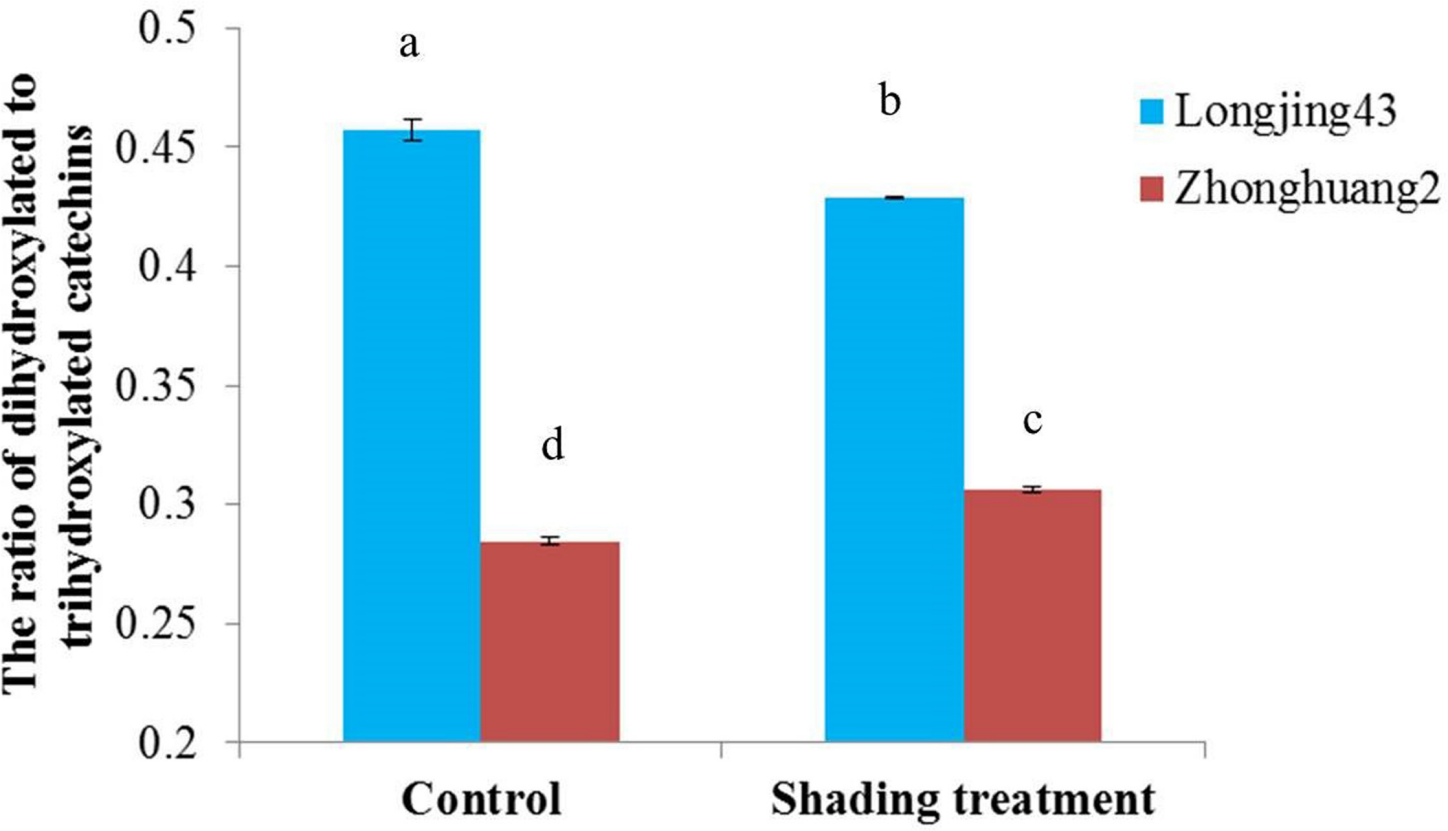
The ratio of dihydroxylated to trihydroxylated catechins in Longjing43 and Zhonghuang2 under control and shading treatment (Mean±standard deviation, n = 3). Means showing significant difference (P < 0.05) are labeled with different letters based on one-way ANOVA with Duncan′s multiple range test.

To further characterize the cultivar and shading effects on RDTC, means and SD of RDTC were calculated and compared. Fig 3 shows that the RDTC in Zhonghuang2 were 38% (control) and 29% (shading treatment) lower than those in Longjing43. While the RDTC of the control and shading treatment showed only 6% (Longjing43) and 8% (Zhonghuang2) differences, which were much lower than the differences between cultivars (Fig 3). Further analysis of variance (ANOVA) showed that the RDTC is mainly affected by cultivars (Partial Eta Squared = 0.999) rather than shading treatment (Partial Eta Squared = 0.412), which is consistent with previous findings [9–13].

### Identification of F3′H and F3′5′H Genes by RNA-seq Analysis

To identify F3′H and F3′5′H genes that may affect dihydroxylated and trihydroxylated catechin biosynthesis, tea samples from cultivars under both control and shading treatment were used for RNA-Seq analysis. Four cDNA libraries were generated and subjected to sequencing by the Illumina HiSeq2000 genome analyzer. A total of 74.7 million 101-bp paired-end reads were obtained (Table 2) and deposited in the National Center for Biotechnology Information (NCBI) with accession number of PRJNA261659 (http://www.ncbi.nlm.nih.gov/bioproject/261659). Low-quality sequences were then trimmed and combined cleaned reads from four libraries were assembled using the software Trinity. Finally, a total of 109,909 unigenes were obtained, with an average length of 793bp and N50 length of 992 bp (Table 3).

**Table 2:** Summary for RNA-Seq datasets of *C*. *sinensis*.

Cultivars	Treatments	Number of reads (million)	Total base (Gb)	Q20 percentage
Longjing43	Control	18.659	3.591	85.15%
	Shading	17.783	3.592	88.77%
Zhonghuang2	Control	18.245	3.685	88.72%
	Shading	20.011	4.042	89.03%

**Table 3:** Length distribution of assembled unigenes.

Unigenes length (bp)	Number of sequences	Percentage
300–500	53196	48.40%
500–1000	34439	31.33%
1000–2000	14603	13.29%
2000+	7671	6.98%
Total number	109909	
Average length	793	
N50 length	992	

**Fig 4:**
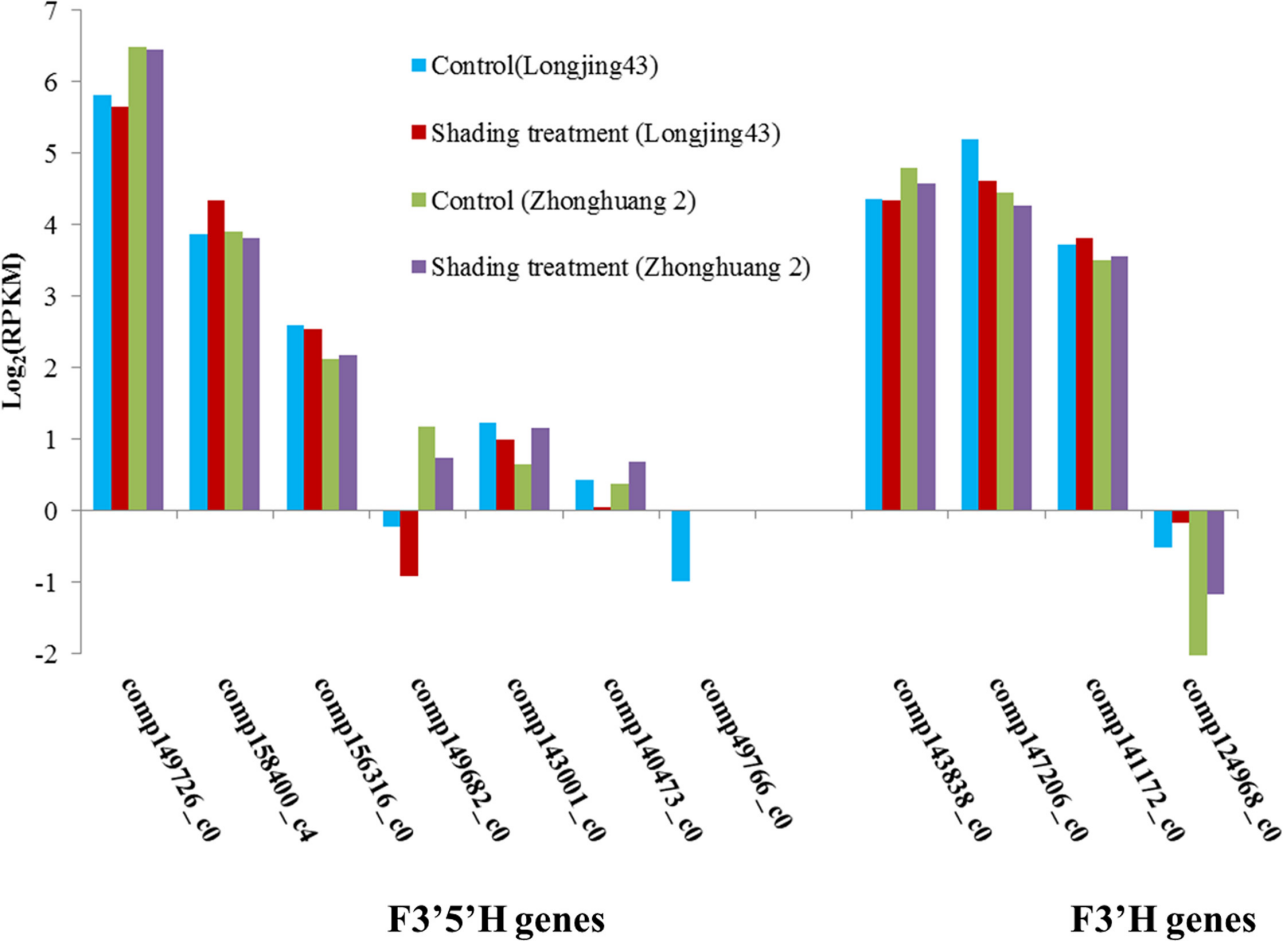
The log_2_(RPKM) of candidate F3′5′H and F3′H genes in Longjing43 and Zhonghuang2 under control and shading treatment.

Thirty six percent of the unigenes (39,982) were annotated using 7 public databases (the COG, GO, KEGG, Swiss-Prot, TrEMBL, NR and NT databases) with an E-value threshold of 10^−5^ (Table 4). Among them, 7 F3′5′H and 4 F3′H genes were identified. Expressions of these unigenes in each library with log_2_(RPKM) were compared in Fig 4. Results showed that the expression of comp149726_c0 (*CsF3′5′H1*) was much higher than other F3′5′H genes, which suggests that it might be the most important F3′5′H gene. Moreover, four F3′5′H genes (comp149682_c0, comp143001_c0, comp140473_c0 and comp49766_c0) had very low log_2_(RPKM), indicating at most 3 F3′5′H genes could be considered as candidate key F3′5′H genes. In terms of F3′Hs, only one unigene (comp124968_c0) showed much lower expression than the other three F3′H genes (*CsF3′H1*, comp143838_c0; *CsF3′H2*, comp147206_c0;*CsF3′H3*, comp141172_c0), which indicates that there might be three key F3′H genes (*CsF3′H1*, *CsF3′H2* and *CsF3′H3*) in *C*. *sinensis*.

**Table 4:** Summary for the BLASTx results of *C*. *sinensis* transcriptome against seven databases.

Annotation database	Annotated Number
COG annotation	9067
GO annotation	26187
KEGG annotation	5877
Swissprot annotation	21325
TrEMBL annotation	34956
NR annotation	34837
NT annotation	29528
All annotated unigenes	39982

Moreover, the RPKM of *CsF3′5′H1* in Zhonghuang2 was much higher, while *CsF3′H2* and *CsF3′H3* were much lower than those in Longjing43, irrespective of treatments, which is consistent with the trend of change in RDTC (Figs 3 and 4). Shading treatment seems to have little effect on RPKM of *CsF3′5′H1*, *CsF3′H1*, *CsF3′H2* and *CsF3′H3* in both cultivars, which explains the less pronounced effects of shading treatment on RDTC.

### Correlation Analysis of Key F3′H and F3′5′H Genes and RDTC

**Fig 5:**
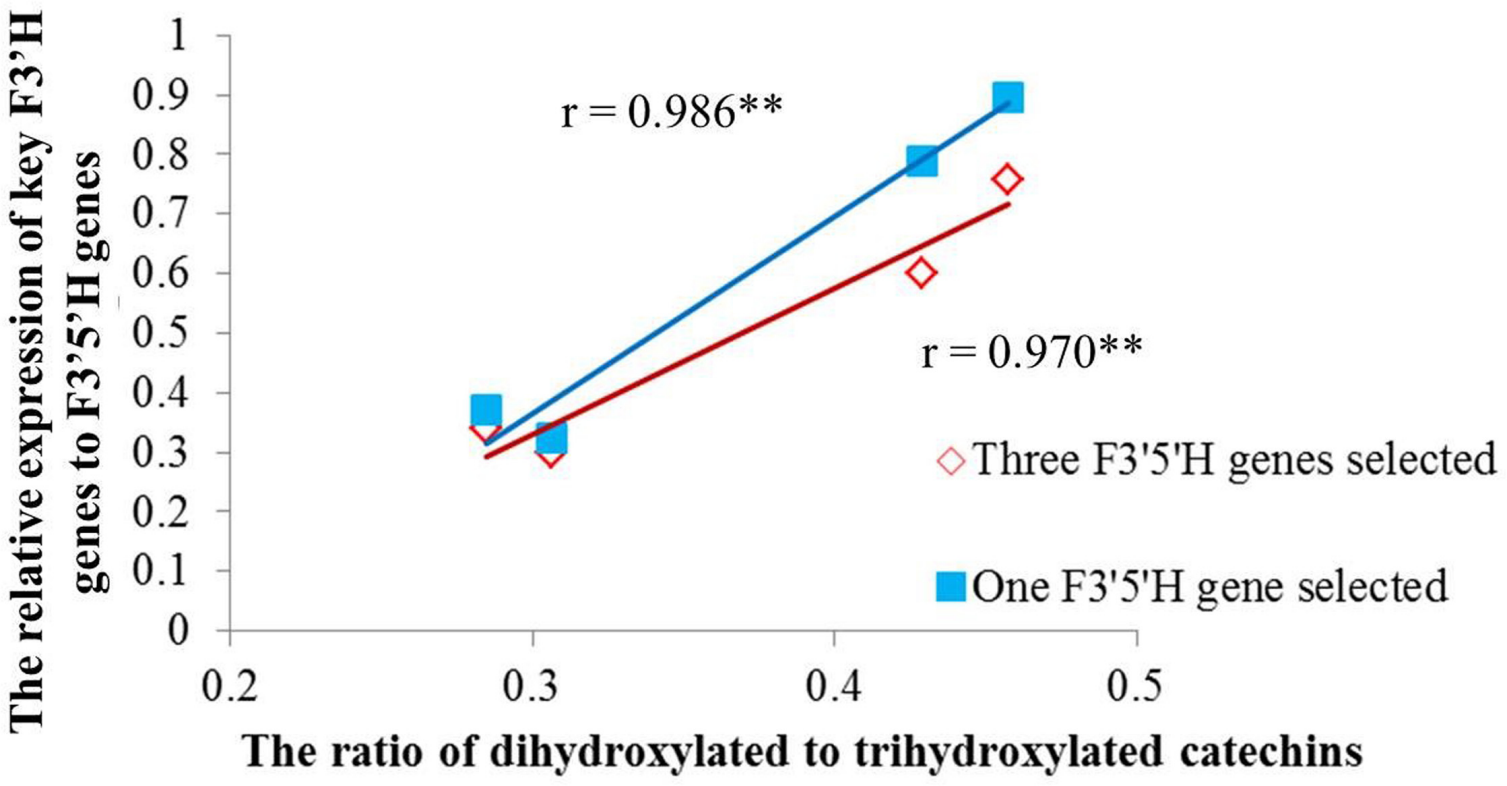
Correlation analysis of the relative expression of key F3′H genes to F3′5′H genes and ratio of dihydroxylated to trihydroxylated catechins in Longjing43 and Zhonghuang2 under control and shading treatment. Red line represents the trend of (*CsF3′H1*+*CsF3′H2*+*CsF3′H3*)/ (*CsF3′5′H1+CsF3′5′H2+CsF3′5′H3*) to the ratio of dihydroxylated to trihydroxylated catechins. Blue line represents the trend of (*CsF3′H1*+*CsF3′H2*+*CsF3′H3*)/ *CsF3′5′H1* to the ratio of dihydroxylated to trihydroxylated catechins. ** represents highly significant at p< 0.01.

Correlation analysis was carried out to further explore the relationship between key F3′H, F3′5′H genes and RDTC (Fig 5). The expression ratios of key F3′H genes (*CsF3′H1*+*CsF3′H2*+*CsF3′H3*) to *CsF3′5′H1* and key F3′H genes to the top three highly expressed F3′5′H genes were calculated to determine their effects on the variation of RDTC. Correlation analysis displayed that both expression ratios were highly significantly correlated with RDTC, indicating the expression ratio of F3′H to F3′5′H genes plays a vital role in determining the variance of RDTC (Fig 5). Furthermore, the correlation coefficient between the expression ratio of key F3′H genes to *CsF3′5′H1* and RDTC (r = 0.986) was a bit higher than that between key F3′H genes to top three F3′5′H genes and RDTC (r = 0.970), suggesting that *CsF3′5′H1* is much more important than other F3′5′H genes in affecting RDTC.

### Phylogenetic Analysis

**Fig 6:**
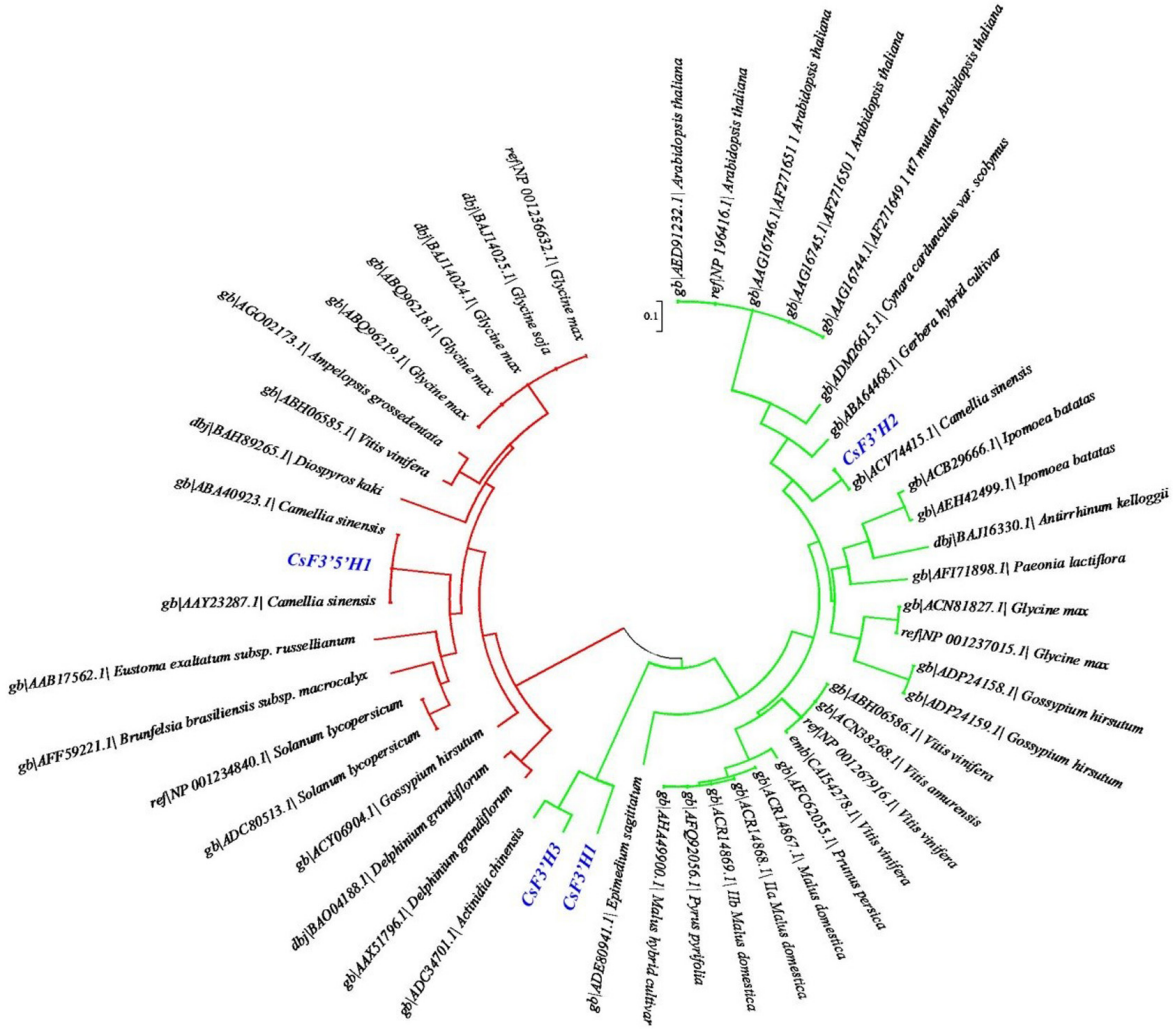
Phylogenetic analysis of F3′H and F3′5′H amino acid sequences. Red lines represent F3′5′H and green lines represent F3′H. Four key genes identified in this study are marked in blue color.

When the deduced amino acid sequences of the four key genes were utilized in a BLAST search of GenBank (http://blast.ncbi.nlm.nih.gov/Blast.cgi), *CsF3′5′H1* and *CsF3′H2* were closely matched to the putative F3'5'H (GenBank accession AAY23287) and F3′H (GenBank accession ACV74415) from *C*. *sinensis*, with 100% and 99% identity respectively. *CsF3′H1* and *CsF3′H3* were highly similar to F3′H from *Actinidia chinensis* (GenBank accession ADC34701), with 66% and 83% identity respectively, indicating that they were newly identified in *C*. *sinensis*. ClustalX was utilized to compare amino acid sequences with 49 F3′H and F3′5′H genes, which include 45 sequences downloaded from the NCBI protein database and 4 key genes identified in this study. A phylogenetic tree was generated according to the alignment results by MEGA5.05 (Fig 6). The F3′H and F3'5'H sequences were classified into two distinct clusters, which suggests they differ in basic structure. Based on the blast search and phylogeny analysis, the *CsF3'5'H1* and three *CsF3′Hs* genes were clearly classified into different gene families, indicating their potentially different functions. Furthermore, *CsF3′H2* seems to be largely different from *CsF3′H1* and *CsF3′H3*. Therefore, their differences in enzyme activities and expression patterns are worth further detail studies.

### Validation of the Relationship Between Key F3′H, F3′5′H Genes and RDTC Among Thirteen Varieties

**Fig 7:**
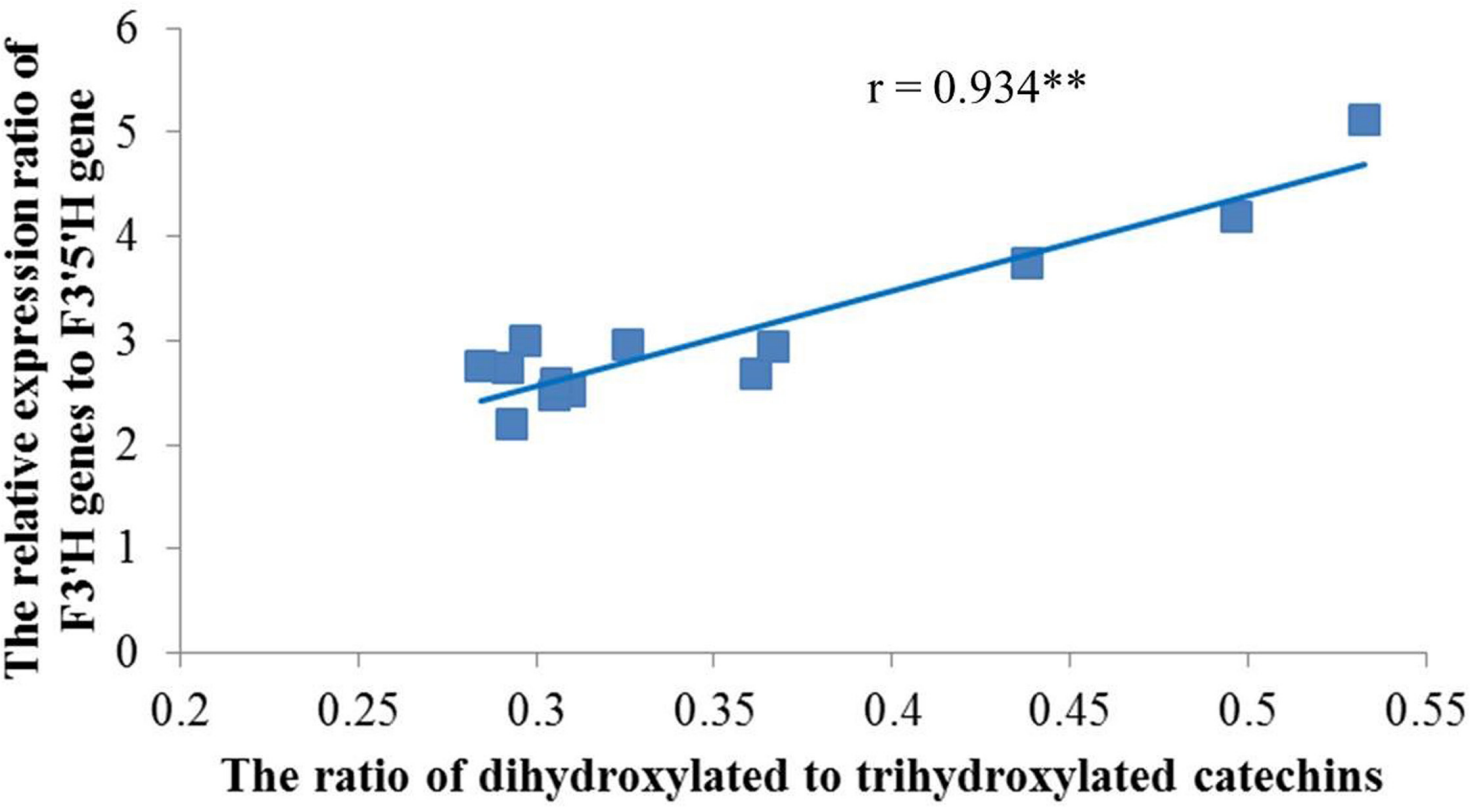
Correlation of the relative expression of key F3′H genes to *CsF3′5′H1* and the ratio of dihydroxylated to trihydroxylated catechins among 13 varieties. Blue line represents the trend of the relative expression of (*CsF3′H1*+*CsF3′H2*+*CsF3′H3*)/ *CsF3′5′H1* to the ratio of dihydroxylated to trihydroxylated catechins. ** represents highly significant at p< 0.01.

To validate the RNA-Seq results, thirteen varieties were selected for real time RT-PCR and catechin analysis. Five of them (Longjing43, Fudingdabaicha, Anjibaicha, Wuniuzao, Zhongcha108) are widely cultivated in China, and the rest were newly bred by TRICAAS. According to our previous research, these varieties showed large differences in RDTC (data not shown). Therefore, both the expressions of key F3′H, F3′5′H genes and RDTC of these varieties were determined in this study to validate their correlation. The results showed that the highest RDTC was identified in XKW15 (0.533) and the lowest in Zhonghuang2 (0.285) (S1 Fig). Furthermore, the RDTC of Longjing43 and Zhonghuang2 harvested in September 2014 were not largely different from those plucked in July 2013, demonstrating that RDTC is also not greatly affected by season. The real time RT-PCR results are shown in S2 and S3 Figs. Correlation analysis unveiled that the expression ratios of F3′H genes (*CsF3′H1*, *CsF3′H2* and *CsF3′H3*) to *CsF3′5′H1* were significantly and positively correlated with RDTC among the thirteen varieties (r = 0.934), which is highly consistent with our previous RNA-Seq results (Fig 7).

## Discussion

Both F3′H and F3′5′H enzymes as well as their encoding genes have been widely studied in horticultural species and fruit trees, such as cyclamen [33], rose [34] and grapevine [35], as they are important in determining flower and fruit coloration. However, for tea plants, understanding of these enzymes is still limited, although they may play an important role in affecting catechin compositions in tea leaves. A novel development is that Wang et al. (2014) firstly confirmed a *CsF3′5′H* (NCBI cDNA accession number: DQ194358) functioned as a F3′5′H enzyme [36]. According to his study, when CsF3′5′H was transformed into *Nicotiana tabacum*, the expressions of CsF3′5′H were positively correlated with the ratio of delphinidin to cyanidin in different transgenic lines. However, whether it is the key gene affecting trihydroxylated catechin biosynthesis in tea leaves is still not clear. This would be largely dependent on its expression level as relative to other homologous genes. Wu et al. (2014) did a correlation analysis between catechin contents and the expressions of their corresponding genes identified by RNA-Seq [22]. According to his study, the high dihydroxylated catechin contents in tea variety ‘Ruchengmaoyecha’ might be associated with the low expression of a F3′5′H gene. However, except for that, few direct correlation analysis was reported between candidate F3′H or F3′5′H genes and RDTC in *C*. *sinensis*. Therefore, a global search of the major F3′H and F3′5′H genes in *C*. *sinensis* is required for relative study.

The current work is the first to show that the coexpression of four key genes (including a F3′5′H gene and three F3′H genes) was closely correlated with the RDTC in tea samples by RNA-Seq and HPLC analysis (Fig 5). Phylogenetic analysis showed the various F3′H and F3′5′H amino acid sequences were clearly classified into two groups (Fig 6), which indicates both enzymes could be distinguished by their amino acid sequences. Moreover, the association of the four key genes and RDTC was further confirmed among 13 tea varieties by real time PCR and HPLC analysis (Fig 7), demonstrating the importance of these four genes in affecting RDTC variation among varieties.

Among the four key genes, *CsF3′5′H1* is the most highly expressed F3′5′H gene in *C*. *sinensis*, which was verified by both RNA-Seq (Fig 4) and real time PCR analysis. According to the blast search, it is 100% identical to the F3'5'H gene from *C*. *sinensis* (AAY23287), a gene highly expressed in young and developing tea leaves, but at most minimally expressed in stems, roots and cotyledons [14]. Furthermore, *CsF3′5′H1* was found to have 96% identity with *CsF3′5′H* (DQ194358) characterized by Wang′s group (2014) according to amino acid sequences [36]. The expression pattern of *CsF3′5′H* (DQ194358) was tissue specific, very high in buds and low in roots, which was similar to AAY23287 [14, 36]. Moreover, the expression of *CsF3′5′H* (DQ194358) was not significantly affected by shading in bud and first leaves, which was similar to our results [32]. Therefore, these results confirmed that *CsF3′5′H1* and *CsF3′5′H* (DQ194358) are homologous genes in different varieties and they are the most important F3′5′H genes in tea buds and leaves.

Although F3′5′H is the key enzyme controlling the formation of trihydroxylated flavonoids [15], few direct correlation between the expression of *CsF3′5′H1* and trihydroxylated catechins has been observed by either the present research or other relative studies, revealing the formation of trihydroxylated catechins might also rely on the expressions of F3′H genes, which are involved in the competition for naringenin and dihydrokaempferol with F3′5′H [32, 37]. Three key F3′H genes (*CsF3′H1*, *CsF3′H2* and *CsF3′H3*) were observed in this study, which was consistent with the finding by Jiang et al. (2013) [38]. Phylogenetic analysis showed that *CsF3′H2* was largely different from *CsF3′H1* and *CsF3′H3*. Meanwhile, the expression of *CsF3′H2* was much higher than *CsF3′H1* and *CsF3′H3* in most varieties (S2 Fig), illustrating *CsF3′H2* may play a more vital role in the formation of dihydroxylated catechins. Furthermore, single nucleotide polymorphism (SNP) markers of *CsF3′H1*, *CsF3′H3* and *CsF3′5′H1* were developed according to the RNA-Seq results (S1 Table), which might be correlated with RDTC. However, no SNP was identified in *CsF3′H2*, indicating the gene might be highly conserved in tea plants. Anyway, further studies are required to verify this hypothesis, such as enzyme activity and Western blot analysis.

It is also worth noting that shading treatment had little effect on RDTC in *C*. *sinensis*, which was attributed to the stable expression of the key F3′H and F3′5′H genes (Figs 3 and 4). Both F3′H and F3′5′H belong to cytochrome P450 protein family and their activities rely on associated proteins like cytochrome P450 reductase and cytochrome b_5_, which are involved in the transfer of electrons to their prosthetic heme group [39]. Bogs et al. (2006) reported a putative cytochrome b_5_ gene was closely correlated with the expression of F3′H and F3′5′H genes in grape [35]. Here, 13 putative cytochrome b_5_ genes were identified in this study. Among them, the expression patterns of two cytochrome b_5_ genes (accession number: KT354240, KT354241) were closely associated with the expressions of F3′H and F3′5′H genes. Further exploration of these genes will facilitate a deeper understanding of the regulation system of the key F3′H and F3′5′H genes in *C*. *sinensis*.

## Conclusions

In summary, four key F3′H and F3′5′H genes were identified to be correlated with RDTC in *C*. *sinensis*. Both RDTC and the expression of key F3′H and F3′5′H genes were mainly affected by varieties rather than shading treatment, which was in agreement with previous findings [9, 10]. Although the regulation system of F3′H and F3′5′H genes in different variety remains elusive, our work provides a direction for future study in this field. Moreover, the transcripts obtained in this study will also facilitate genomic studies on *C*. *sinensis*.

## Supporting Information

S1 Fig The variation of ratio of dihydroxylated to trihydroxylated catechins in 13 tea varieties (Mean±standard deviation, n = 3). (TIF)

S2 Fig Real time-PCR analysis of key F3′H genes in 13 tea varieties. Data of real time PCR analysis are the means and standard deviations (n = 4).
(TIF)

S3 Fig Real time-PCR analysis of *CsF3*′*5*′*H1* genes in 13 tea varieties. Data of real time PCR analysis are the means and standard deviations (n = 4).
(TIF)

S1 Table SNP markers of *CsF3′H1*, *CsF3′H3* and *CsF3′5′H1*. (XLSX)

## List of Abbreviations

AE: Amplification efficiency
ANOVA: Analysis of variance
C: (+)-catechin
COG: Clusters of Orthologous Groups of proteins
EC: (-)-epicatechin
ECG: (-)-epicatechingallate
EGC: (-)-epigallocatechin
EGCG: (-)-Epigallocatechingallate
F3′H: Flavonoid 3′-hydroxylase
F3′5′H: Flavonoid 3′,5′-hydroxylase
GC: (-)-gallocatechin
GO: Gene ontology
KEGG: Kyoto Encyclopedia of Genes and Genomes
NR: Non-redundant protein
NT: Nucleotide collection
RDTC: Ratio of dihydroxylated to trihydroxylated catechins
RPKM: Reads per kilobase per million reads
SD: Standard deviations
