# Supplementary figures and images for: Transcriptome Analysis Reveals Key Flavonoid 3′-Hydroxylase and Flavonoid 3′,5′-Hydroxylase Genes in Affecting the Ratio of Dihydroxylated to Trihydroxylated Catechins in Camellia sinensis
Source: PLoS One. 2015 Sep 14;10(9):e0137925. doi: 10.1371/journal.pone.0137925 (PMC4569414; doi:10.1371/journal.pone.0137925)

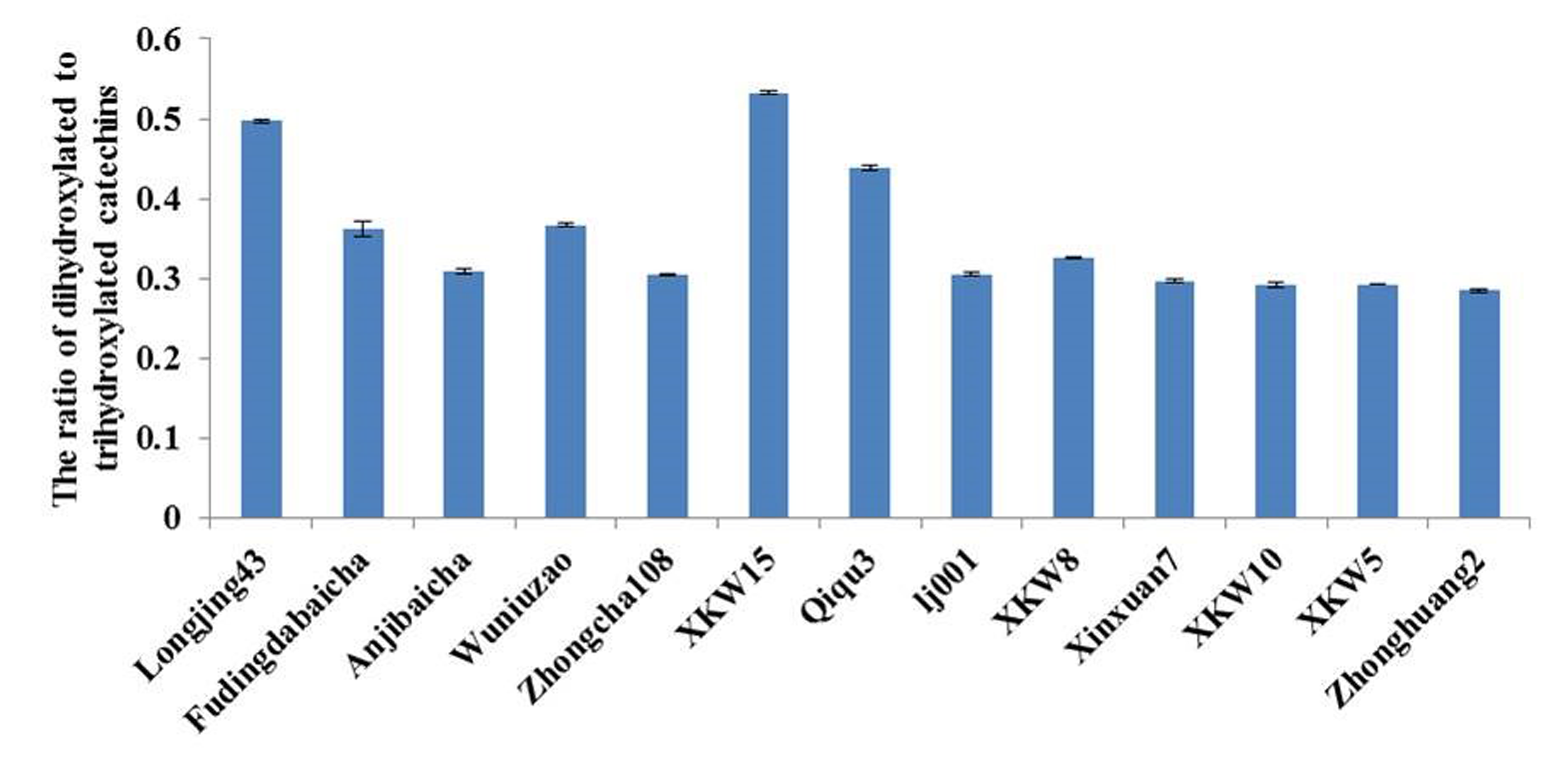

Supplement: S1 Fig — (TIF) [file pone.0137925.s001.tif]

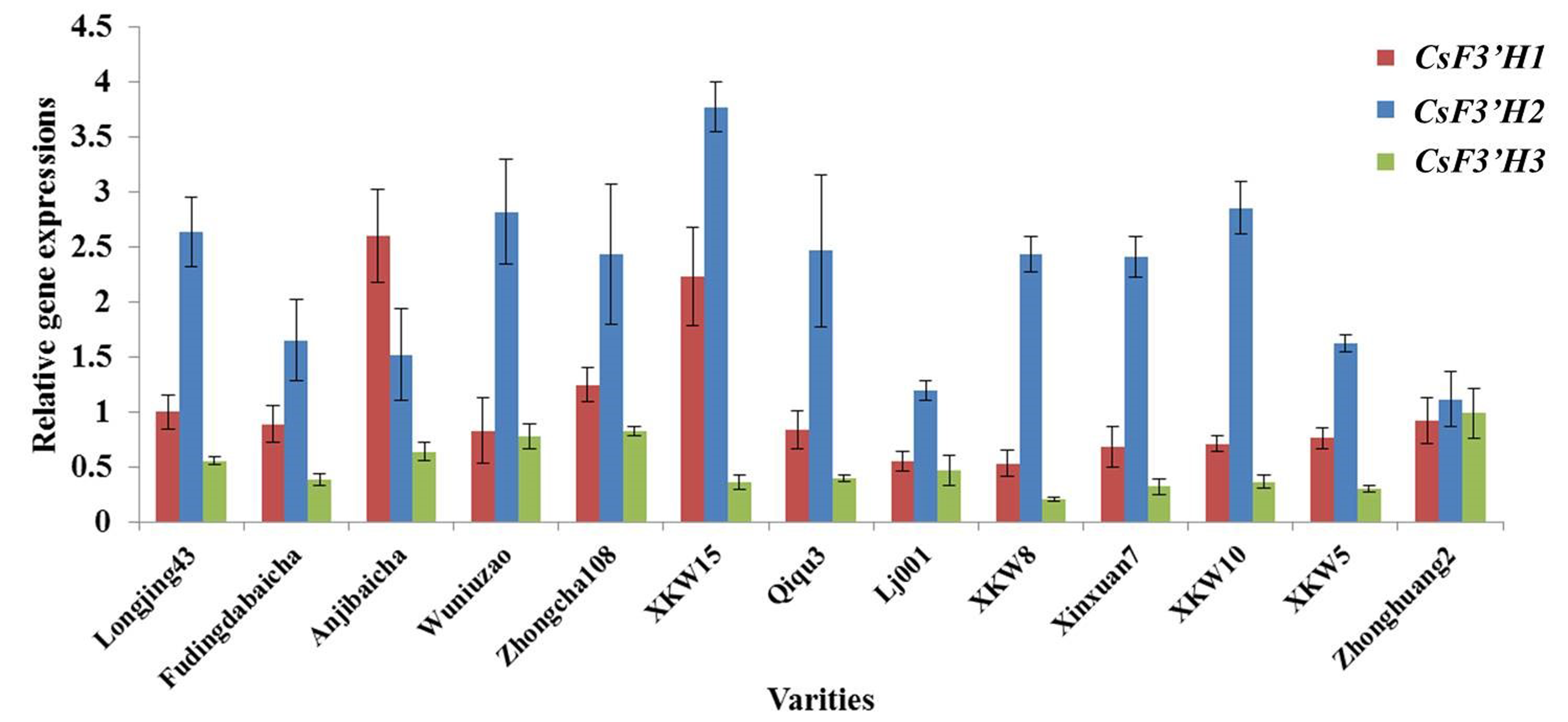

Supplement: S2 Fig — Data of real time PCR analysis are the means and standard deviations (n = 4). (TIF) [file pone.0137925.s002.tif]

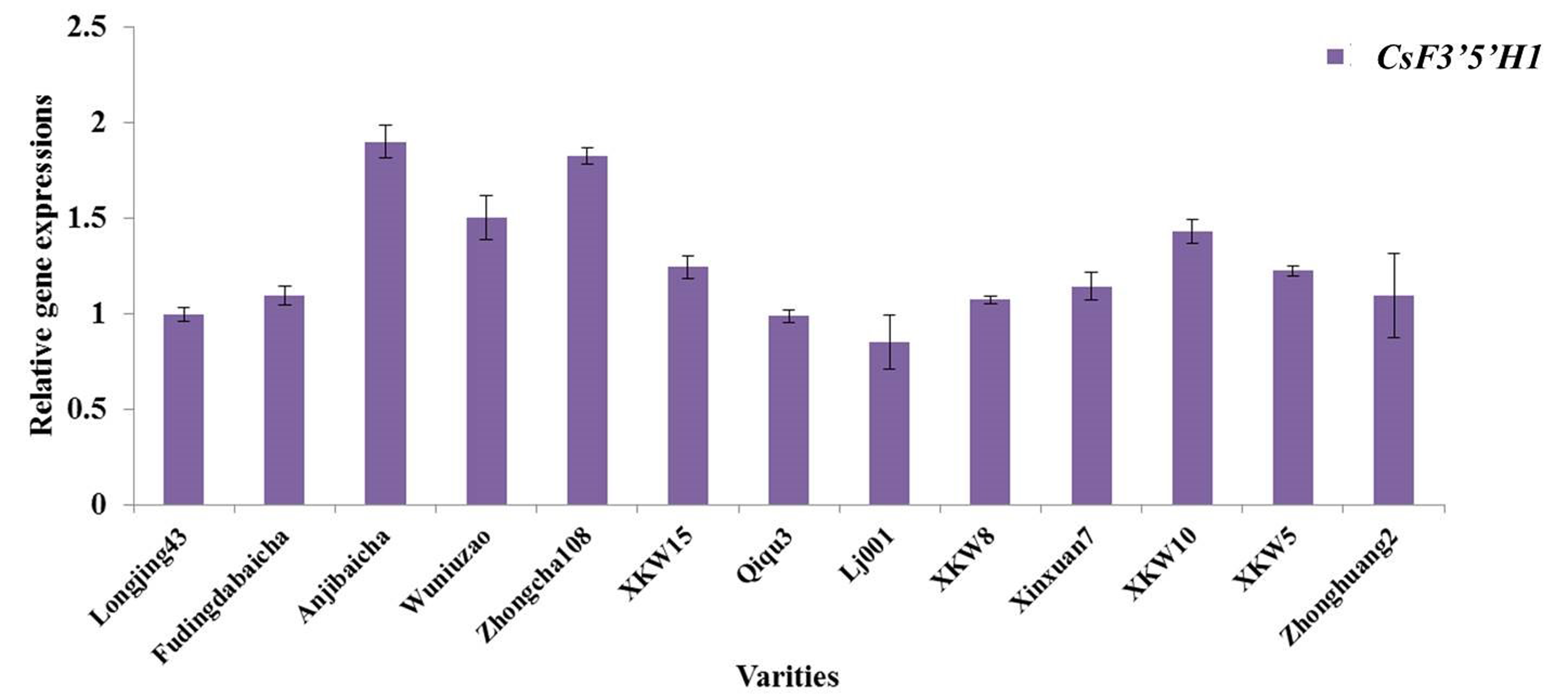

Supplement: S3 Fig — Data of real time PCR analysis are the means and standard deviations (n = 4). (TIF) [file pone.0137925.s003.tif]
